# Recent advances in the management of transient ischemic attacks

**DOI:** 10.12688/f1000research.12358.1

**Published:** 2017-10-26

**Authors:** Camilo R. Gomez, Michael J. Schneck, Jose Biller

**Affiliations:** 1 Department of Neurology, Loyola University Medical Center, Maywood, IL, USA

**Keywords:** transient ischemic attack, TIA, stroke risk stratification, antiplatelet therapy, anticoagulants, arterial revascularization

## Abstract

Significant advances in our understanding of transient ischemic attack (TIA) have taken place since it was first recognized as a major risk factor for stroke during the late 1950's. Recently, numerous studies have consistently shown that patients who have experienced a TIA constitute a heterogeneous population, with multiple causative factors as well as an average 5–10% risk of suffering a stroke during the 30 days that follow the index event. These two attributes have driven the most important changes in the management of TIA patients over the last decade, with particular attention paid to effective stroke risk stratification, efficient and comprehensive diagnostic assessment, and a sound therapeutic approach, destined to reduce the risk of subsequent ischemic stroke. This review is an outline of these changes, including a discussion of their advantages and disadvantages, and references to how new trends are likely to influence the future care of these patients.

## Introduction

**Figure 1.**
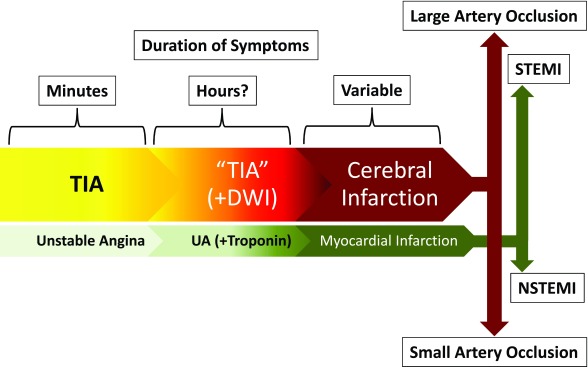
Comparison of the anatomoclinical relationship between transient ischemic attack (TIA) and cerebral infarction with the acute coronary syndromes. TIAs can be considered the equivalent of unstable angina, with symptoms lasting for a few minutes and then abating. They often have a longer duration and an imaging counterpart (positive diffusion weighted imaging [DWI]), which may be reversible. When the ischemic process is sufficiently severe, it results in permanent injury to the brain tissue. This "ischemic continuum" mirrors the findings in acute coronary syndromes, even to the point that cerebral infarctions resulting from large arterial occlusions are emergently managed endovascularly, just like an ST-elevation myocardial infarction (STEMI), while the smaller infarctions are handled by non-interventional means, just like a non-STEMI (NSTEMI).

The original description of TIA as a clinical entity dates back to a 1958 report by C. Miller Fisher, in which he described it as cerebral ischemic episode that "... may last from a few seconds up to several hours, the most common duration being a few seconds up to five or 10 minutes…" ^1^. Our understanding of TIAs has increased since then, with a growing realization that they represent a warning about the risk of ischemic stroke, an ominous prelude to an impending cerebrovascular catastrophe, but also the opportunity to prevent a disabling event ^2– 5^. The current definition of TIA precludes its use as a diagnosis in patients whose imaging studies display acute ischemic tissue injury (i.e. infarction), irrespective of the resolution of their symptoms ^6^. This shift from a phenomenologic to an anatomoclinical view may seem frivolous, but it underscores the importance of TIA as an opportunity for stroke prevention, much like unstable angina heralds myocardial infarction (MI) ( Figure 1) ^7^. This similarity is the backdrop for our discussion of the most important advances in the management of TIAs: a) improved predictive models of stroke risk (i.e. stroke risk stratification), b) optimal algorithms for evaluation (i.e. comprehensive assessment), and c) effective treatment strategies (i.e. therapeutic approach).

## Stroke risk stratification

### Etiopathogenic variability

Unlike unstable angina, TIAs can result from very different causative factors, each with its own particular risk profile ^8, 9^. Therefore, the care of patients with TIA requires etiopathogenic evaluation and individualized estimation of their stroke risk. It seems intuitive that if a) TIA is to be considered the warning (i.e. a "threat" or "alarm") of an impending ischemic stroke and b) its assigned stroke risk depends on its pathogenic process, prevention strategies should match stroke subtype. Stroke subtype classification is best carried out by applying the scheme designed for the Trial of ORG-10172 in Acute Stroke Treatment (TOAST) ^8^. Unfortunately, the likelihood of correctly identifying stroke subtype in the emergency department (ED) approximates 60%, making it impractical for use before patients are fully evaluated ^10^.

Thus, although the risk of stroke following a TIA is estimated at 5–10%, and despite 15–20% of ischemic stroke patients reporting a premonitory TIA, their etiopathogenic heterogeneity and inherent variability of stroke risk is illustrated by each patient profile ^4, 11– 13^. This has led to the development of various scoring systems for stroke risk stratification ^2– 5, 7, 11, 12, 14, 15^, all striving to satisfy the following attributes: a) ability to discriminate between high and low stroke risk, b) consistency of performance across clinical scenarios ^15^, and c) rapid applicability, since time is of the essence in the ED ^2, 3, 5, 7, 11, 12, 14^.

### Stroke risk scoring systems

The scoring systems from the 1990's were concerned with long-term stroke risk ^16– 21^. Later, increasing appreciation of TIA as a medical emergency led to interest in quantifying short-term risk and early stroke prevention ^3, 22– 30^. The first wave of scoring systems, including the California Risk Score (CRS) ^3^, ABCD ^30^, and ABCD ^2^ ^27^, comprised stroke risk factors (i.e. age, blood pressure, and diabetes mellitus) and semiologic variables (i.e. clinical features and duration of symptoms) ( Table 1). The CRS showed progressively higher scores to be associated with an increasing proportion of stroke events adjudicated during a 90-day follow up period but did not include a final stratification into a high- and low-risk dichotomy ^3^. The ABCD (i.e. age, blood pressure, clinical features, and duration of symptoms) score adjudicated no stroke events with ABCD scores <4, while the 7-day risk of stroke increased to 35.5% with ABCD scores >6 ^30^. In another population, ABCD scores <2 were associated with no 30-day stroke events, the risk progressively increasing with higher scores, reaching 31.3% in patients with ABCD scores of 6 ^31^. The most significant finding was an 8-fold increase with ABCD ≥5 ^31^, suggesting the presence of a "tipping point" at which stroke risk increases exponentially. The introduction of the ABCD score was rapidly embraced by the community of vascular neurologists and has provided the seminal platform for the introduction of progressively more complex scoring systems. The combination of the CRS and the ABCD scores resulted in the ABCD ^2^ score, which stratified three different risk groups as low (<4), moderate (4–5), and high (6–7) risk ^27^.

**Table 1.** Comparison of the different stroke risk scoring systems used in patients with transient ischemic attack (TIA). See text and Figure 2 for additional information. CIP, clinical imaging-based prediction; CRS, California Risk Score; DBP, diastolic blood pressure; DWI, diffusion weight imaging; SBP, systolic blood pressure.

System	Semiologic Variables	Risk Factors	Imaging Findings	Score	% Stroke Risk (Period)
CRS (2000) ^3^	Duration >10 minutes Weakness Speech Impairment	Age >60 Diabetes	N/A	1 2 3 4 5	3 7 11 15 34 (90 day)
ABCD (2005) ^30^	Weakness Speech Duration	Age >60 SBP >140 mmHg or DBP >90 mmHg	N/A	1–3 4 5 6	0 2.2 16.3 35.5 (7 day)
ABCD2 (2007) ^27^	Weakness Speech Duration	Age >60 SBP >140 mmHg or DBP >90 mmHg Diabetes	N/A	1–3 4–5 6–7	1.0 4.1 8,1 (2 day)
ABCD2 + MRI (2007) ^23^	Weakness Speech Duration	Age >60 SBP >140 mmHg or DBP >90 mmHg Diabetes	+DWI	1–4 5–6 7–9	0 5.4 32.1 (90 day)
CIP (2009) ^22^	Weakness Speech Duration	Age >60 SBP >140 mmHg or DBP >90 mmHg Diabetes	+DWI	>3 +DWI >3 & +DWI	2.0 4.9 14.9 (7 day)
ABCD2-I (2010) ^26^	Weakness Speech Duration	Age >60 SBP >140 mmHg or DBP >90 mmHg Diabetes	+DWI or +CT	1–2 3 4 5 6 7 8 9 10	0 1.2 1.6 2.0 3.7 4.0 12.3 15.1 12.2 (7 day)
ABCD3 (2010) ^28^	Weakness Speech Duration	Age >60 SBP >140 mmHg or DBP >90 mmHg Diabetes Recent TIA	N/A	1–3 4–5 6–9	1 2 6 (90 day)
ABCD3-I (2010) ^28^	Weakness Speech Duration	Age >60 SBP >140 mmHg or DBP >90 mmHg Diabetes Recent TIA	+DWI or carotid stenosis	1–3 4–7 8–13	1 2 8 (90 day)
ABCDE⊕ (2010) ^25^	Weakness Speech Duration Etiology	Age >60 SBP >140 mmHg or DBP >90 mmHg Diabetes	+DWI	1–3 4 5 6 7 8 9 10 11 12	0 7 15 3 17 20 28 24 18 25 (90 day)

However, the predictability of these scores was not optimal and they only partially fulfilled the criteria outlined earlier. Concurrently, abnormal diffusion weighted imaging (DWI) on magnetic resonance imaging (MRI) was found to correlate with stroke risk ^32, 33^. Thus, the addition of a DWI-positive point to the ABCD ^2^ score resulted in a system whose tiers had different 90-day stroke risks (i.e. 0% for scores <4 and up to 32.1% for scores of 7–9) and, additionally, different 90-day risks for functional impairment, an outcome not measured by the original ABCD ^2^ score ^23^. Imaging-related stratification improvement has been replicated using two other systems, the clinical imaging-based prediction (CIP) model ^22^ and the ABCD ^2^-I ^26^.

The next wave of scoring systems improved the predictability of stroke risk by including diagnostic information as it became available ^28^. The ABCD ^3^ added the occurrence of a TIA within the preceding week to the ABCD ^2^ score, but the results were disappointing ^28^. However, the same group added two more imaging variables (i.e. DWI lesions and carotid stenosis) to the ABCD ^3^ model, showing that ABCD ^3^-I was superior to previous scores in predicting stroke at 7, 28, and 90 days ^28^. A similar approach to stroke risk stratification involved the Alberta Stroke Prevention in TIAs and Mild Strokes (ASPIRE) intervention, which assigned a high-risk denomination to patients with a) ABCD ^2^ scores ≥4, b) motor or speech symptoms lasting >5 minutes, or c) atrial fibrillation (AF) ^24^. However, ASPIRE allocated over three-quarters of patients to the high-risk category, cancelling out the benefit of triaging TIA patients.

The most recent initiative involves the addition of "etiology" to an algorithm that already includes all the variables listed above ^25^. The ABCDE⊕ scoring system was designed by adding two variables to the ABCD score: a) "etiology", which included a point-weighting of each particular stroke subtype based on published data ^34^, and b) "DWI-positivity", which consisted of arbitrarily assigning three points to a positive MRI based on inferential conclusions from published data ^25, 35^. Setting an arbitrary boundary of >6 points to designate high-risk patients, this system compared favorably with both ABCD ^2^ (i.e. statistically significant) and ABCD (i.e. trend only) in identifying TIA patients at high risk for stroke ( Table 1) ^25^.

### Perspective and practical approach to stroke risk prediction

**Figure 2.**
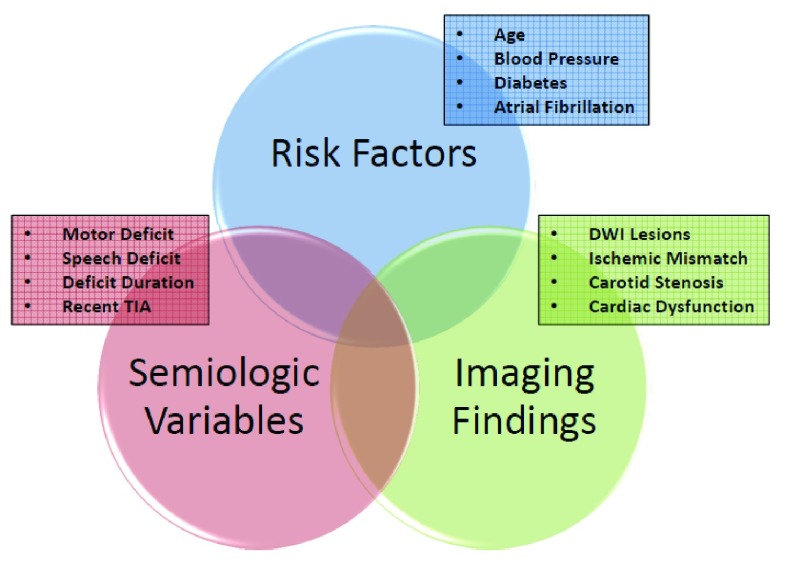
Variables that compose the different stroke risk scoring systems. Three domains are used to group the components contained within the different stroke risk scoring systems. The earlier systems (i.e. California Risk Score [CRS], “age, blood pressure, clinical features, and duration of symptoms” [ABCD], and ABCD ^2^) only included components from the “risk factors” and “semiologic variables” domains. The more recent ones (i.e. ABCD ^2^-MRI, clinical imaging-based prediction [CIP], ABCD ^2^-I, ABCD ^3^-I, and ABCDE⊕), shown to have better predictability, added one or more components from the “imaging findings” domain.

Stroke risk stratification of patients with TIA, reliably identifying those more likely to benefit from urgent intervention, is a subject of considerable importance in vascular neurology. At the bedside, it is useful to approach stroke risk stratification by systematically considering the three domains that include the variables that comprise the scoring systems described ( Figure 2): a) risk factors (i.e. age, hypertension, diabetes, AF, and previous TIA), b) semiologic variables (i.e. type of symptoms and signs and duration of deficit), and c) imaging findings (i.e. DWI lesions, carotid stenosis, and etiology).

In applying a domain-driven approach to stroke risk assessment, it is possible to simultaneously address the components of all of the scoring systems described without actually being bound by any one of them at the expense of others. It can be argued that the disadvantage is that it may not be possible to equate every single clinical scenario with a numerical prediction of stroke risk. On the other hand, arriving at a numerical representation of the stroke risk does not seem to be as important as identifying which patients have a greater than minimal risk, a clinical task that requires the expert formulation of a more qualitative view of the patient as an individual ^36– 43^. This approach seems supported by existing experience in comparing the simultaneous application of the various stroke risk scoring systems. Recent reports by a European consortium of vascular neurology investigators, the "Proyecto Español del Manejo y Evaluación de los Pacientes con un Ataque Isquémico Transitorio" (PROMAPA), underscore the importance of going beyond the scoring systems, particularly in unstable patients and those with recurrent TIAs ^29, 44^.

The limitation of the various scoring systems is not surprising when considering that a) the boundaries between the various levels of stroke risk have been set arbitrarily, b) some variables are better defined than others (e.g. blood pressure is defined as >140 mmHg systolic and/or >90 mmHg diastolic but, at which point? How many measurements does it take?), and c) the point weighting of some variables (e.g. imaging) has been derived from previous, often not replicated studies. In addition, in many clinical scenarios where TIA patients are seen for the first time, much of the information required for scoring diagnostic variables is not available, and only the simpler of the scoring systems are applicable.

## Comprehensive assessment

The diagnostic evaluation of TIAs revolves around the determination of the cause and mechanism of the index event. In addition, as TIA is a medical emergency worthy of urgent management, diagnostic investigations require an efficient and expedited algorithm.

### Timing and location

A major topic of debate involves when, where, and how to evaluate TIA patients most effectively. The typical patient presents *after* the symptoms of the index event have abated, creating two conflictive considerations: a) the inconvenience and discomforts of hospitalizing a neurologically normal patient and b) the inherent risks of a protracted ambulatory evaluation ^45– 50^. Opinions regarding the best approach are sharply divided ^51^, with the proponents of an ambulatory workflow citing "cost-effectiveness" and better "resource utilization" as their main arguments ^47, 52, 53^. Conversely, those who favor an in-hospital process argue for "expediency of care", "patient safety", and better outcomes ^46, 48, 53, 54^. The most recent literature on this subject is, at best, inconclusive; both approaches have advantages and disadvantages based on two sets of variables: a) the patient's stroke risk profile and b) the clinical environment construct and capabilities. The former has been covered in the previous sections, but the latter is worthy of further discussion.

Traditionally, TIA patients present to the ED because of extensive educational campaigns consistently instructing them to "DIAL 9-1-1" upon recognition of stroke symptoms. They are then admitted for "23-hour observation" while their evaluation is completed. The benefits of this approach include a) patients have a captive audience of medical personnel who watch over them in case of a neurologic change, b) the results of the diagnostic tests are known almost immediately and can be used in real time to update the stroke risk calculation, c) the identification of a need for urgent therapeutic intervention allows rapid execution of any treatment plan, and d) there is little risk of suffering an ischemic stroke while waiting to have the tests completed (including results review) or to "fall through the cracks" because of scheduling mishaps. Experts agree that this approach is appropriate when managing high-risk TIA patients ^54^ but question whether it is justified for those at low risk ^47, 51^. Unfortunately, low scores do not necessarily translate into low stroke risk, particularly in the ED. In fact, approximately 20% of patients with ABCD ^2^ <4 harbor either an atherosclerotic or a cardiogenic source of stroke and 3-month stroke risk comparable to those with scores >4 ^37^.

**Figure 3.**
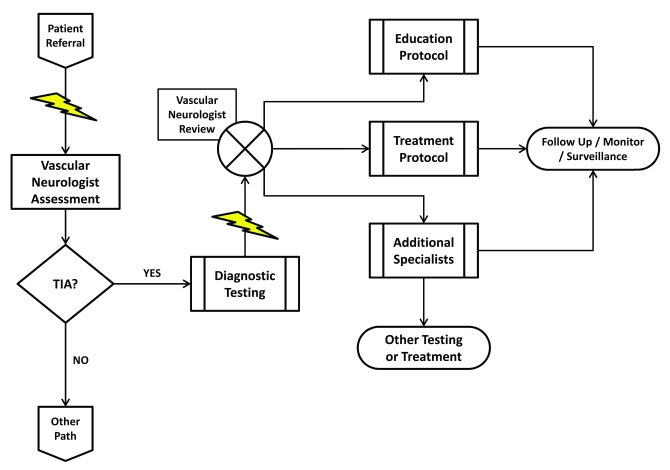
Optimal workflow in a transient ischemic attack (TIA) clinic. A potential TIA patient is referred to the clinic and his visit with the vascular neurologist is expedited (lightning bolt). Upon assessment, if the patient is found not to have had a TIA, his care is diverted to an alternative clinical pathway. If the vascular neurologist determines that the patient has had a TIA, he can proceed by immediately requesting the appropriate diagnostic procedures. Test completion and results reporting are also expedited (lightning bolt), allowing the vascular neurologist to quickly review them and decide on a treatment strategy tailored to the patient's risk profile. Management includes patient and family education as well as appropriate referral to pre-specified specialists.

A recently introduced alternative environment for the evaluation of TIA patients is the TIA clinic ( Figure 3) ^51, 55– 57^, whose demonstrated effectiveness and beneficial impact on outcome ^57, 58^ depend on the following attributes:

a) Fast track access: referral of potential TIA patients must unequivocally result in immediate appointments ^51, 53– 57^. It is unreasonable to consider TIA as an emergency and simultaneously subject the patient to the inherent delays of ambulatory care. Thus, the established metrics are appointments made within 24 hours for high-risk patients and within 48 hours for others ^59– 61^.

b) Specialist (i.e. vascular neurologist) assessment: there is simply no substitute for experience. The diagnosis of TIA can be challenging ^62, 63^, as many other conditions may "mimic" its presentation ^64– 71^. Moreover, its identification must be followed by a cerebrovascular localization diagnosis, which has a direct impact on the etiopathogenic assessment and stroke subtype diagnosis ^72^. Only a specialist in cerebrovascular disorders can rightfully prioritize the diagnostic and therapeutic needs of a TIA patient ( Figure 3) ^73, 74^.

c) Rapid access to diagnostic investigations: once a patient is evaluated by a specialist, diagnostic investigations must be carried out very quickly ( Figure 3). Such a workflow can be challenging, particularly when competing for time slots or when, in the case of transesophageal echocardiography (TEE), another specialist's participation is required. Once completed, the vascular neurologist must have rapid access to the results in order to be able to make the next set of decisions ( Figure 3).

d) Multidisciplinary network: there must be access to a variety of consultants from other disciplines, particularly cardiologists, neurosurgeons, and vascular surgeons. This implies that these specialists are also available at a moment's notice to evaluate the patient ( Figure 3).

e) Educational programs: patients must be educated in relevant topics, such as stroke risk factors and their management, beneficial lifestyle changes, and interventions (i.e. medications and procedures) used in the prevention of stroke.

### Depth of diagnostic evaluation

The battery of diagnostic tests required for TIA evaluation is geared at uncovering the cause and mechanism (i.e. stroke subtype classification) of the index event. Most TIA patients fall into either the large artery atherothromboembolic (~30–40% of cases) or the cardiogenic (~15–20%) categories (i.e. TOAST) ^75^, the two subtypes that pose the greatest stroke risk following TIA ^34^. Therefore, the priorities of such diagnostic evaluation involve examination of the cerebral arterial system ^76, 77^ and a search for cardiogenic sources of embolization ^78– 86^. Other tests (e.g. coagulation studies), although certainly important, are easier to carry out, only infrequently lead to positive findings, and rarely carry a sense of urgency.

The cerebral vasculature can be evaluated noninvasively with magnetic resonance angiography (MRA), computed tomography angiography (CTA), or neurovascular ultrasound (i.e. extracranial carotid and vertebral color Doppler and transcranial Doppler ultrasound). The choice of which to use depends on a) the clinical scenario, b) the specific vascular pathology suspected, and c) the imaging resources available at any particular clinical site. One advantage of MRA is that it can be completed concurrently with MRI, which is the stroke imaging technique of choice ^35, 75, 76, 87– 92^ and a component of several stroke risk scoring systems ^22– 26^. However, CTA has also been shown to be useful in the early assessment of TIA ^93^ as well as for patients who cannot undergo MRI. Patients with renal insufficiency who cannot receive contrast (either iodinated or gadolinium-based) can often only be evaluated using ultrasound. When noninvasive cerebrovascular imaging is inconclusive or when it indicates pathology that requires intervention, cerebral catheterization and angiography remains an important tool for evaluation and treatment selection ^94, 95^. Although cerebral angiography is invasive and certainly with some risks, the information obtained from this technique is often not available by any other means.

As for cardiogenic TIA, the introduction of TEE represents a major diagnostic advancement due to a) increased resolution and sensitivity for left atrial pathology, including the left atrial appendage and the interatrial septum, and b) capability for imaging the aortic arch, uncovering the presence of complex atherosclerotic plaques as a source of artery-to-artery cerebral embolism ^80– 82, 84, 85^. Finally, the detection of occult paroxysmal AF in patients with unexplained events correlates with the length of the cardiac rhythm assessment ^86^, improved by 7- to 30-day cardiac event monitors ^96– 101^ and even more so by implantable loop recorders ^83, 97– 99, 101^.

## Therapeutic strategies

### General measures

Despite the significant risk of stroke during the first week following a TIA ^3, 4, 12, 102^, stroke prevention can be optimized (i.e. stroke risk reduction of ~80%) by rapid and intensive interventions ^57, 58, 103^. Undoubtedly, tailoring therapeutic measures to the specific etiopathogenic mechanism (i.e. stroke subtype) is the most desirable strategy. However, since such assessment cannot be made with certainty in the ED ^10^, the immediate management of patient presentation should be viewed as part of a continuum, with certain specific measures (e.g. antiplatelet therapy) possible with minimal risk and potentially significant benefit as soon as the first CT scan is reviewed ^104– 107^.

Some general measures of care don't even require any imaging to be completed for their implementation. For example, isotonic crystalloid solutions can easily be administered to TIA patients as soon as they arrive in the ED. Not only is this inexpensive and low-risk step in line with the existing guidelines for the early management of acute ischemic stroke ^108^ but it also addresses the fact that approximately 50% of stroke patients present with measurable dehydration ^109– 115^. Along the same lines, any patient with TIA should be given "best medical management" of oxygenation, careful blood pressure control, and serum glucose regulation, just as if they had suffered a *bona fide* ischemic stroke ^108^.

### Target-specific measures

***Antiplatelet therapy*.** Early antithrombotic therapy leads to about 80% relative reduction of stroke risk in patients with TIA, although the best strategy continues to be a matter of debate ^57, 58^. In general, aspirin has been thought to reduce the odds of a subsequent stroke by approximately 20–25% in patients with previous TIA or stroke ^116^ and doses of 50–325 mg per day continue to be recommended due to their lower risk of hemorrhagic side effects ^117^. There is increasing evidence, however, that aspirin is the key interventional step in reducing the early risk of stroke following TIA, with yields of 60% overall relative risk reduction and 70% reduction of disabling or fatal strokes ^103^. In parallel, the Fast Assessment of Stroke and Transient Ischemic Attack to prevent Early Recurrence (FASTER) trial provided evidence that aspirin plus clopidogrel may be substantially beneficial in the hyperacute treatment of patients with TIA ^118^. Similar results were reported by the Clopidogrel with Aspirin in Acute Minor Stroke of Transient Ischemic Attack (CHANCE) study ^119^, with benefit that persisted at one year of follow up ^120^. Another ongoing trial with a similar aim, the Platelet-Oriented Inhibition in New TIA and Minor Ischemic Stroke (POINT) study, has already recruited about 80% of its target sample size and results should be available within 18–24 months ^121^. Patients with large artery atherosclerosis ^8^ have been shown by several studies to benefit from double antiplatelet therapy ^118, 122, 123^. That said, more is not necessarily better, and the recently reported (not yet published) Triple Antiplatelets for Reducing Dependency after Ischemic Stroke (TARDIS) study failed to show a benefit of adding dipyridamole to aspirin and clopidogrel for stroke risk reduction ^124, 125^. The results of TARDIS are not surprising since, although shown to be beneficial in combination with low-dose aspirin ^126, 127^, dipyridamole has not been found to be superior to clopidogrel in reducing the risk of stroke ^128^.

In the last few years, there has been considerable interest in the study of other antiplatelet agents for secondary stroke prevention ^129– 133^. Cilostazol, an agent similar to dipyridamole, has been shown in two Asian studies to be superior to aspirin in reducing vascular events in patients with stroke, with the caveat that those results may not be applicable to other populations. The SOCRATES study showed ticagrelor, a PY2 inhibitor similar to clopidogrel, to be approximately 30% more effective than and equally as safe as aspirin in reducing major adverse events in patients with TIA and atherosclerotic stenosis ^129– 132^.

***Anticoagulants*.** Vitamin K antagonists, namely warfarin, have been in use for many years, and their main applications have been in patients with AF, mechanical heart valve prostheses, and other causes of cardiogenic brain embolism. In patients with non-valvular AF (NVAF), warfarin reduces the risk of stroke by approximately 67% in comparison to no treatment and by 37% when compared with antiplatelet therapy ^134^. The indications for warfarin in other scenarios of cardiac dysfunction are less clearly supported by the literature but reasonable to consider on an individual basis: a) severe left atrial enlargement (LAE), particularly those with spontaneous echo contrast ^135– 143^, b) abnormally low flow in the left atrial appendage (LAA) ^144– 147^, and c) left ventricular (LV) dysfunction ^148– 153^.

Recently, two other classes of oral anticoagulants for the treatment of patients with NVAF have become available ( Table 2): direct thrombin (i.e. dabigatran) ^154– 158^ and factor Xa (i.e. apixaban ^159– 164^, rivaroxaban ^165^, and edoxaban ^166, 167^) inhibitors. These medications have all been successfully tested against warfarin in patients with NVAF, and their appeal is based on the fact that they have shorter half-lives, fewer drug and food interactions, and do not require laboratory therapeutic monitoring ^154– 167^. Presently, only dabigatran has a specifically designated reversing agent (i.e. idarucizumab – Praxbind®) ^168^, a fact that adds a unique dimension to its use. Unfortunately, the use of these new anticoagulants is not without its own set of problems. For instance, they are all excreted via the kidneys, so care must be exercised in their use in patients with renal insufficiency ^160^.

**Table 2.** Comparison of the principal trials that have studied the new generation of anticoagulants. See text for more detailed information. ARISTOTLE, Apixaban for Reduction in Stroke and Other Thromboembolic Events in Atrial Fibrillation; AVERROES, Apixaban Versus Acetylsalicylic Acid to Prevent Stroke in Atrial Fibrillation Patients Who Have Failed or Are Unsuitable for Vitamin K Antagonist Treatment; BID, bis in die; CHADS2, congestive heart failure, hypertension, age ≥75 years, diabetes mellitus, stroke (double weight); ENGAGE AF, Effective Anticoagulation with Factor Xa Next Generation in Atrial Fibrillation; RE-LY, Randomized Evaluation of Long-Term Anticoagulation Therapy; ROCKET AF, Rivaroxaban Once Daily Oral Direct Factor Xa Inhibition Compared with Vitamin K Antagonism for Prevention of Stroke and Embolism Trial in Atrial Fibrillation; TIA, transient ischemic attack.

Drug	Dabigatran (110 mg BID)	Dabigatran (150 mg BID)	Apixaban (5 mg BID)	Apixaban (5 mg BID)	Rivaroxaban (20 mg daily)	Edoxaban (60 mg daily)
**Trial**	RE-LY ^154^	RE-LY ^154^	AVERROES ^159^	ARISTOTLE ^161^	ROCKET-AF ^165^	ENGAGE-AF ^166^
**Mean age (years)**	71.4	71.5	70	70	73	72
**Mean CHADS2**	2.1	2.2	2.0	2.1	3.5	2.8
**Previous** **TIA/stroke (%)**	20	20	14	19	55	28.1
**Mean time in target** **range (%)**	64	64	N/A	62	58	68.4
**Rate of stroke/** **embolism (%/year)**	1.5 v. 1.69	1.11 v. 1.69	1.6 v. 3.7	1.27 v. 1.60	1.7 v. 2.2	1.18 v. 1.50
**Rate of major** **bleeding (%/year)**	2.71 v. 3.36	3.11 v. 3.36	1.4 v. 1.2	2.13 v. 3.09	3.6 v. 3.4	2.75 v. 3.43
**Mortality from any** **cause (%/year)**	3.75 v. 4.13	1.9 v 2.2	3.5 v. 4.4	3.52 v. 3.94	1.9 v. 2.2	3.99 v. 4.35

Successful anticoagulation of patients with AF depends on careful benefit versus risk assessment. This is aided by available scoring systems, namely the CHA _2_DS _2_-VASc score ^169, 170^ (i.e. a "second generation" improvement of the original CHAD _2_ score ^171^) to determine stroke risk and the HAS-BLED score to quantify risk of hemorrhagic complications ( Table 3) ^172^. Scoring allows characterization of yearly risks along stepwise progressive scales, which can then be easily compared for the purposes of clinical decisions ^169, 170, 172^. Typically, calculation of the HAS-BLED score requires more attention to detail, since its components have been more precisely defined ^172^. For example, while in CHA _2_DS _2_-VASc the "H" stands for "hypertension" as an active diagnosis ^169^, in the HAS-BLED the "H" stands for "uncontrolled hypertension" (i.e. >160 mmHg systolic) ^172^. Moreover, the original definition does not specify over what period, but it seems reasonable that, in order to contribute to the bleeding risk, the blood pressure would have to be persistently elevated. Consequently, not only is a single measurement insufficient to assign a point but, practically, aggressive blood pressure control should reduce the HAS-BLED score ( Table 3).

**Table 3.** Comparison of the two scoring systems most commonly used to assess benefit versus risk of anticoagulation in patients with non-valvular atrial fibrillation. HAS-BLED definitions ^171^: Hypertension = *"'Uncontrolled hypertension' as evidenced by >160 mmHg systolic."* Abnormal renal function = *"Chronic dialysis, renal transplantation, or serum creatinine ≥200 μmol/L"* Abnormal liver function = *"Chronic hepatic disease (e.g. cirrhosis) or biochemical evidence of significant hepatic derangement (e.g. bilirubin >2Xupper limit normal, and so forth."* Stroke = *"Previous history, particularly lacunar"* Bleeding history or predisposition = *"Any bleeding requiring hospitalization and/or causing a decrease in haemoglobin level >2 g/L and/or requiring blood transfusion that was not a hemorrhagic stroke"* Labile international normalized ratio (INR) = *"Therapeutic time in range <60%"* Elderly = *">65 years"* Drugs = *"Antiplatelet agents, non-steroidal anti-inflammatory drugs"* Alcohol excess= *"≥8 units alcoholic consumption per week"* AP, angina pectoris; LVD, left ventricular dysfunction; MI, myocardial infarction; PVD, peripheral vascular disease; TE, thromboembolism; TIA, transient ischemic attack.

CHA2DS2-VASc ^169^		HAS-BLED ^172^	
Criteria [POINTS]	Stroke & TE Risk (% Yearly)	Criteria [POINTS]	Bleeding Risk (% Yearly)
**C**ongestive heart failure/LVD [1]	1...................1.3	**H**ypertension [1]	1...................1.02
**H**ypertension [1]	2...................2.2	**A**bnormal renal [1] or liver [1]	2...................1.88
**A**ge ≥ 75 years [2]	3...................3.2	**S**troke [1]	3...................3.74
**D**iabetes mellitus [1]	4...................4.0	**B**leeding [1]	4...................8.70
**S**troke/TIA/TE [2]	5...................6.7	**L**abile INRs [1]	5...................12.5
**V**ascular disease (MI, PVD, AP) [1]	6...................9.8	**E**lderly [1]	
**A**ge 65–74 years [1]	7...................9.6	**D**rugs [1] or alcohol excess [1]	
**S**ex **c**ategory (female) [1]	7...................6.7		
	9...................15.2		

***Arterial revascularization*.** There is a robust body of knowledge to indicate that patients who have experienced a TIA due to an ipsilateral carotid atherosclerotic stenosis of more than 50% benefit from revascularization either by carotid endarterectomy (CEA) ^173, 174^ or by carotid artery stenting (CAS) ^174– 177^. Either procedure should be carried out as soon as it is practical, preferably within two weeks of the index event ^174, 178^, by expert teams, in centers that perform a high volume of procedures, and with a track record of a major complicating event rate of <6% ^174, 178^.

In patients with TIA and extracranial vertebral artery stenosis, despite the existing literature ^179– 181^, the guidelines suggest that stenting be reserved for patients who remain "symptomatic" despite optimal medical therapy, including risk factor modification and antithrombotic agents ^174^. The recommendation for patients with intracranial atherosclerosis, even those with severe stenosis, is that they are managed with maximal medical therapy ^174^, based on the results of the Stenting and Aggressive Medical Management for Preventing Recurrent Stroke in Intracranial Stenosis (SAMMPRIS) study ^182, 183^. Although it is beyond the scope of our review to undermine the SAMMPRIS study, we must indicate the number of criticisms made of its design and execution ^184– 188^ and suggest that additional study of this subject is needed.

***Vascular risk factor modification*.** Blood pressure control is recommended to reduce the risk of stroke in patients with TIA ^174^. The desirable blood pressure is uncertain in the few hours that follow the index event, when a "normal" level may aggravate ischemia because of abnormal autoregulation or upstream pressure gradients. Subsequently, blood pressure should be reduced to <140/90 mmHg in most patients and <130/80 mmHg in diabetics ^174^.

Intensive lipid-lowering therapy with statins should be instituted in patients with atherosclerosis and low-density lipoprotein cholesterol (LDL-C) >100 mg/dL ^174^. Moreover, early use of statins has been shown to benefit patients with TIA without causing undue adverse events ^189– 191^, although not uniformly (e.g. the FASTER study failed to show any benefit from simvastatin) ^118^. An exciting prospect is the introduction of proprotein convertase subtilisin/kexin type 9 (PCSK9) inhibitors which, when added to statins, seem to result in a more effective reduction of LDL-C ^192, 193^, with potential beneficial effect in stroke risk reduction ^194– 199^.

Diabetes and the metabolic syndrome have been associated with an increased risk of stroke ^200^. Patients with TIA should be screened for diabetes by means of hemoglobin A1c (HbA1c) and for obesity by measuring the body mass index (BMI) ^174^. In the future, it may be possible to predict insulin resistance in non-diabetic TIA patients by means of scoring systems ^201^. Recent data suggest that abnormal glycemic control may also have a negative effect on the efficacy of clopidogrel ^202^, and glucose reduction through diet, exercise, oral hypoglycemic agents, and insulin to a fasting <126 mg/dL is recommended ^174^. The pharmacologic choices for achieving glucose control are numerous and their application depends on different clinical considerations. However, in the recently published Insulin Resistance Intervention after Stroke (IRIS) study, pioglitazone was shown to reduce the risk of stroke and MI by approximately 24% in patients with recent TIA ^203^.

In addition to all of the previous recommendations, patients must be counselled about smoking cessation, proper diet (preferably Mediterranean), regular exercise, maintenance of appropriate BMI, and limiting alcohol consumption as measures that require their own active participation to reduce their risk of subsequent stroke ^174^.

## Conclusion

The diagnosis of a TIA represents the recognition of a medical emergency and an opportunity to reduce the risk of stroke by decisively evaluating the patient and applying any combination of the currently available therapeutic strategies. The future is likely to show additional methods of early diagnosis, better algorithms for stroke risk stratification, and enhanced systems of care for these patients, without a dependence on hospitalization.
